# White and brown adipose tissue functionality is impaired by fine particulate matter (PM_2.5_) exposure

**DOI:** 10.1007/s00109-022-02183-6

**Published:** 2022-03-14

**Authors:** Lucio Della Guardia, Andrew C. Shin

**Affiliations:** 1 Department of Biomedical Sciences for Health, Università Degli Studi Di Milano, via Fratelli Cervi 93, 20090 Segrate, Milano, Italy; 2 Department of Nutritional Sciences, College of Human Sciences, Texas Tech University, Lubbock, TX USA

**Keywords:** Adipocytes, Air pollution, Inflammation, Insulin resistance, Metabolic diseases, Macrophages

## Abstract

Chronic exposure to high levels of particulate matter (PM) is correlated to a higher prevalence of cardio-metabolic disturbances. Adipose tissue represents a pivotal regulator of metabolic homeostasis, and its dysfunction is associated with health issues in PM-exposed models. This review discusses the adaptive changes of white (WAT) and brown (BAT) adipose tissue in response to fine particulate matter (PM_2.5_), investigating the underlying pathophysiology. In exposed models, PM_2.5_ increases oxidative stress and impairs mitochondria functionality and biogenesis in WAT and BAT. Chronic exposure also upregulates the main apoptotic/pro-inflammatory pathways and promotes the infiltration of monocytes and the accumulation of activated macrophages. Oxidative stress and inflammation are responsible for the inhibition of insulin signal transduction and glucose uptake in both the adipose tissues. The increased inflammatory status also suppresses the metabolic activity of brown adipocytes, promoting the *whitening*. Altogether, this evidence suggests the shift of WAT and BAT toward an inflammatory and metabolic dysfunctional phenotype. Although the underlying mechanisms remain to be clarified, the development of inflammation in lungs, gut, and hypothalamus seems to have a pivotal role in the alteration of adipose tissue homeostasis. The potential consequences on systemic cardio-metabolic health render the relationship PM-adipose tissue a key issue to investigate.

**Graphical abstract:**

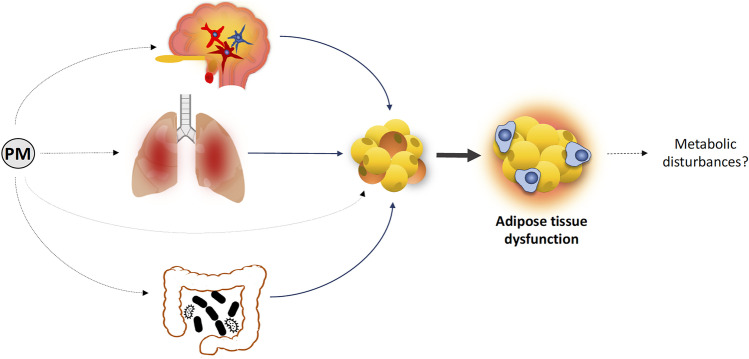

## Introduction

Particulate matter (PM) is a component of air pollution containing a complex mixture of solid and liquid particles, derived from human activities and natural sources. Variability in size, shape, and chemical composition is responsible for differing toxicity of inhaled PM [1]. Chronic exposure to airborne PM has a profound impact on human health, especially in the early stages of life and frail subjects [2, 3]. In humans and animals, the chronic inhalation of fine PM (PM_2.5_; mean diameter of particles ~2.5 μm) is associated with the development of insulin resistance [4], metabolic syndrome [5], and diabetes [2, 6–8]. The functional impairment of tissues such as the brain, liver, and adipose tissue, secondary to PM_2.5_ exposure, is likely implicated in the development of such dysmetabolic conditions [9–11].

Growing evidence suggests that PM_2.5_ inhalation negatively influences both white and brown adipose tissue (WAT and BAT, respectively) [12, 13]. In addition, the development of dysfunction in both the adipose tissues is associated with the worsening of health conditions in humans and animals undergoing chronic PM exposure [12, 14–28].

Considering these findings, the investigation of adipose tissue response to PM_2.5_ can represent a crucial step for understanding the health consequences of prolonged PM_2.5_ exposure. This review discusses the adaptive and pathological responses of BAT and WAT of rodents undergoing PM_2.5_ exposure.

## Adipose tissue is an important regulator of metabolic homeostasis

Adipose tissue is an ensemble of different cell types comprising adipocytes, immune, vascular, and stromal cells. Adipocytes express a highly adaptive biological profile [29] as they can activate specific pathways in response to surrounding environmental changes and varying nutritional conditions [29, 30]. The crosstalk between adipose tissue and organs such as the brain, liver, and skeletal muscle helps to coordinate an articulated network, which is critical for the control of systemic metabolic health [30–32].

WAT is primarily intended to store the energy surplus in the form of triglycerides (TGs) [30]. The tight inter-communication between adipocytes and resident immune cells regulates inflammatory balance and insulin sensitivity in WAT [30, 32–34]. Factors such as energy excess, toxicants, and pro-inflammatory agents can disrupt this equilibrium leading to the development of inflammation and metabolic dysfunction [30]. The spillage of pro-inflammatory/diabetogenic mediators such as interleukin 1β (IL-1β), interleukin 6 (IL-6), tumor necrosis factor-α (TNFα), and C–C motif chemokine ligand-2 (CCL2) from dysfunctional WAT worsens gluco-metabolic health [35–37].

BAT promotes the conversion of stored fats into energy through the sympathetic-mediated activation of uncoupling protein (UCP)-1 [34, 38]. Brown adipocytes increase energy expenditure and improve insulin sensitivity by stimulating glucose and fatty free acids (FFAs) oxidation [34, 38, 39]. White and brown adipocytes can mutually interconvert given appropriate stimuli [40]: physical activity stimulates the *browning* [34, 40]; in contrast, obesity is correlated with increased *whitening* [38, 41]. The *whitening* of brown adipocytes is characterized by decreased mitochondrial fitness and marks dysfunction as white-shifted adipocytes are prone to develop a pro-inflammatory/apoptotic phenotype [34, 42]

## Airborne particulate matter

Airborne PM is a collection of microscopic particles of different sizes, consisting of carbonaceous particles with adsorbed chemicals [43]. Transition metals (Fe, Cu, Ni) account for the larger fraction of inorganic molecules constituting PM. Endotoxins, polycyclic aromatic hydrocarbons (PAHs), and quinones [44, 45] typically represent the organic fraction of PM. Differences in size, shape, and chemical composition are responsible for the toxicity of specific subclasses of inhaled PM [1, 43].

In the respiratory compartment, PM dissolves in the aqueous lining, coming into direct contact with alveolar cells [1]. Alveolar macrophages sequester particles of various size shifting towards an activated phenotype [46]; the PM-induced activation of macrophages is critical for the increase of local and systemic inflammation [1, 47–49].

The finest fraction of PM (~0.2 μm) enters systemic circulation by crossing the alveolar (or gastrointestinal; GI) barrier and could be internalized in tissues via endocytosis-mediated mechanisms [1, 46, 47, 49–53]. Larger particles can reach extrapulmonary organs transported by alveolar macrophages [53]. Also, organic/inorganic fractions of PM, as well as materials adsorbed to the surface of inhaled particles can pass into circulation and accumulate in central/peripheral tissues [43].

## PM_2.5_ exposure increases oxidative stress and impairs mitochondria and endoplasmic reticulum

**Fig. 1:**
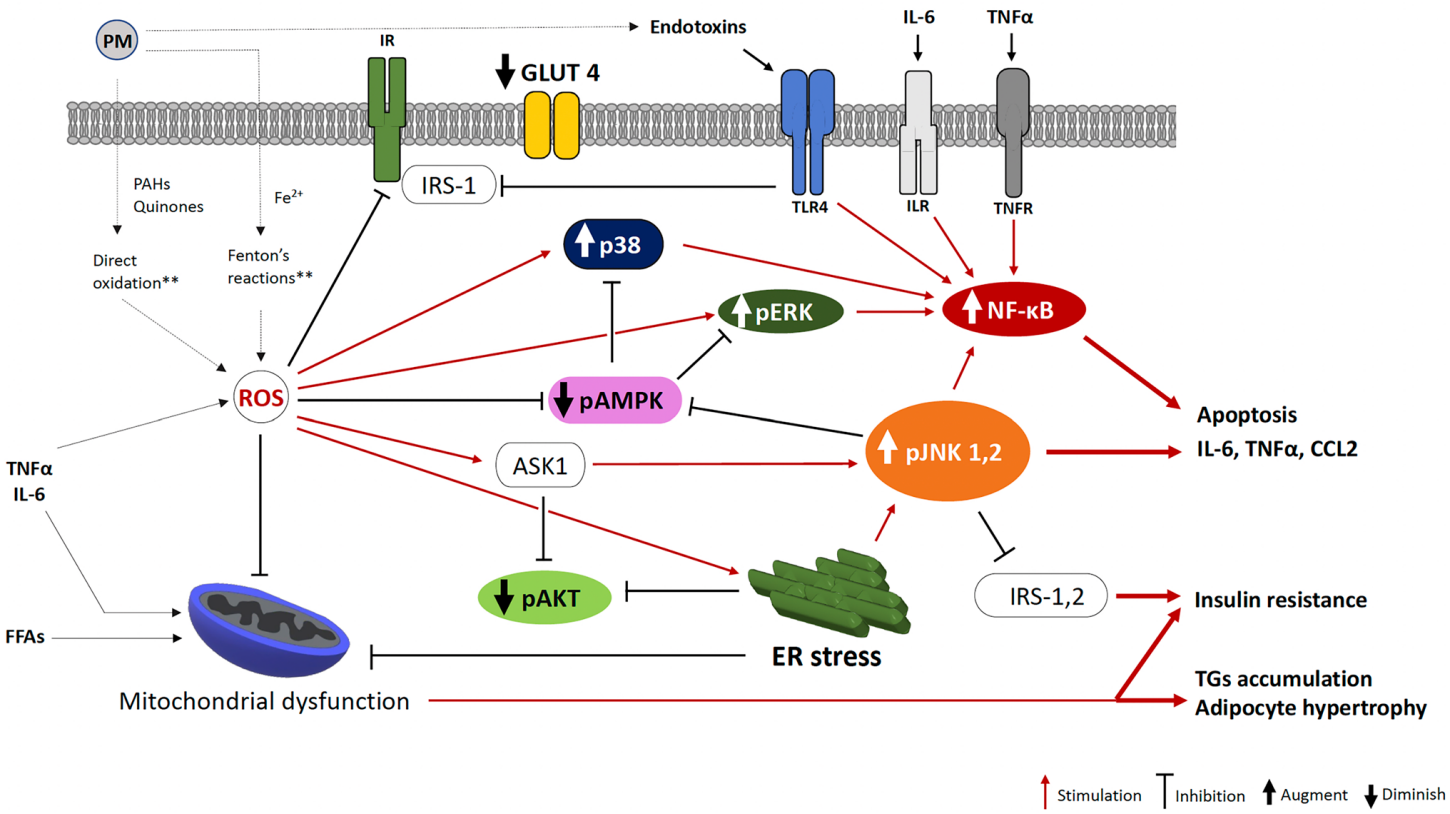
Intracellular pathways conducive to inflammation and insulin resistance are upregulated in adipose tissue of PM_2.5_-exposed animals. PM_2.5_ enhances ROS production. PM_2.5_ constituents (e.g., endotoxins) and pro-inflammatory cytokines stimulate the NF-κB pathway. ROS are responsible for the impairment of ER and mitochondria. ER stress and ROS upregulate JNK, p38, and ERK and suppress AMPK. NF-kB and JNK enable pro-inflammatory/apoptotic response and downregulate insulin signaling. Mitochondrial dysfunction impairs substrate oxidation and insulin sensitivity. AKT, Protein kinase B; AMPK, AMP-activated protein kinase; ASK, apoptosis signal-regulating kinase 1; CCL2, chemokine C–C motif ligand 2; ER, endoplasmic reticulum; ERK, extracellular signal-regulated kinase; IL-6, interleukin 6; IR, insulin receptor; ILR; interleukin receptor; IRS, insulin receptor substrate; JNK 1, 2; NF-κB, nuclear factor kappa-light-chain-enhancer of activated B cells; p38, p38 mitogen-activated protein kinase; PM, particulate matter; ROS, reactive oxygen species; TLR4, Toll-like receptor 4; TGs, triglycerides; TNFα, tumor necrosis factor-α; TNFR, tumor necrosis factor receptor. **These pathways are not demonstrated in adipose tissue

In isolated cells, PM elicits its cytotoxic activity by altering membrane stability and stimulating the production of reactive oxygen species (ROS) [1]. Both the carbonaceous nuclei and adsorbed organic/inorganic chemicals play a critical role in this respect [43]. Reactive constituents of PM (metals, PAHs, quinones) propel the production of ROS by inducing Fenton’s reactions and/or stimulating the oxidation of other organic macromolecules (Fig. 1) [4, 5]. PM can also generate ROS by interfering with the activity of the mitochondrial respiratory chain [54].

The upregulation of superoxide anions and markers such as superoxide dismutase (SOD), nuclear factor erythroid 2-related factor (Nrf)-2, and heat shock 70 kDa protein-1 (HSP72) provides supporting evidence for increased ROS in WAT and BAT of PM_2.5_-exposed animals [23, 55]. Chiefly, HSP72 represents a hallmark of functional derangement under PM exposure, since its expression correlates with inflammation and insulin resistance [56, 57]. ROS act as co-factors in the induction of mitochondria and endoplasmic reticulum (ER) functional impairment [58].

Mitochondria are essential for energy production. In adipose tissue, they regulate lipid metabolism, insulin sensitivity, and the secretion of key adipokines [59, 60]. The proper functioning of mitochondria in WAT and BAT warrants systemic gluco-metabolic homeostasis [34, 42, 61, 62].

PM_2.5_ and its chemical constituents can impair mitochondria [53]. Also, pro-inflammatory mediators and ER stress can negatively impact mitochondrial functionality (Fig. 1) [9, 23, 55, 61–63]. Studies in rodents demonstrate that mitochondrial function and biogenesis are compromised upon prolonged PM_2.5_ exposure [1, 54, 64] (Fig. 1). In WAT and BAT of mice undergoing either inhalation or intratracheal instillation of PM_2.5_, the expression of mitochondrial biogenetic markers–peroxisome proliferator activated receptor gamma coactivator 1-(PGC-1)α and UCP-1–was significantly suppressed [23, 25, 28, 65, 66]. Similarly, a reduction in mitochondrial number and size was observed in BAT and WAT of PM_2.5_-exposed rodents [23, 65, 66]. These changes were associated with the increase in superoxide anion and Nrf-2 in both WAT and BAT [23, 65], suggesting a causal role of ROS in the alteration of mitochondrial biogenesis/functionality and vice versa (Fig. 1).

ER represents a pivotal structure for adipocyte metabolic health [67, 68]. ER dysfunction is responsible for the development of chronic inflammation in adipose tissue [68]. ROS enhances the unfolded protein response (UPR), which represents a cellular self-protection mechanism and a reliable indicator of ER dysfunction [58, 69, 70].

In mice exposed to chronic PM_2.5_ inhalation, Mendez et al. observed a significant increase in ER stress in WAT, as demonstrated by the induction of the UPR activator–binding immunoglobulin protein (BiP) [71]. In addition, the long-term exposure to high PM_2.5_ concentrations was found to activate the UPR pathway by upregulating the expression of regulated IRE1-dependent mRNA decay and the constitutive elements of ER-associated degradation pathways [71].

## PM_2.5_ triggers inflammation and insulin resistance

In WAT of PM_2.5_-exposed animals, mitochondrial and ER impairment is associated with insulin resistance and inflammation [23, 65, 71]. This finding is in accordance with evidence from non-exposed models [61, 68] and indicates that mitochondrial and ER dysfunction can upregulate the molecular pathways conducive to inflammation and insulin resistance in the adipose tissue of PM_2.5_-exposed animals [72] (Fig. 1).

JNK is a cell regulator, enabled by a variety of stressors [73]; its activation induces apoptosis and suppresses the insulin signal in adipocytes [73–75]. PM_2.5_ exposure was demonstrated to augment JNK expression in organs critical for metabolic regulation such as liver and adipose tissue [17, 25, 76]. Pan et al. showed a significant increase in pJNK in the visceral WAT (vWAT) of both lean and *ob/ob* mice undergoing PM_2.5_ inhalation, compared to non-exposed [25].

The activation of p38 mitogen-activated protein kinase (p38) and extracellular signal-regulated kinase (ERK) inhibits insulin signaling [77, 78] and promotes the *whitening* of brown adipocytes [79, 80]*.* In human observational studies, the expression of p38 and ERK in vWAT was correlated with the concentration of PM [81]. In lean and *db/db* mice PM_2.5_ inhalation increased p38 and ERK expression [25]. Interestingly, in high-fat diet (HFD)-fed PM_2.5_-exposed rodents, the inhibition of monocyte recruitment efficiently prevented the activation of p38 in WAT, suggesting that the immune-mediated inflammation is involved in the activation of the p38 pathway [18].

The 5′-AMP-activated protein kinase (AMPK) enables the translocation of the glucose transporter (GLUT)-4 to plasma membrane and promotes glucose and FFAs oxidation [82]. In WAT, AMPK suppresses the nuclear factor kappa-light-chain-enhancer of activated B-cells (NF-κB) and the adipocyte pro-inflammatory shift [17, 25, 83, 84] (Fig. 1). In animal models, PM_2.5_ intratracheal instillation significantly reduced phosphorylated AMPK (pAMPK) in the subcutaneous WAT (sWAT) of lean mice [28]. In contrast, the pharmacological activation of AMPK effectively prevented the PM_2.5_-induced *whitening* in BAT.

**Fig. 2:**
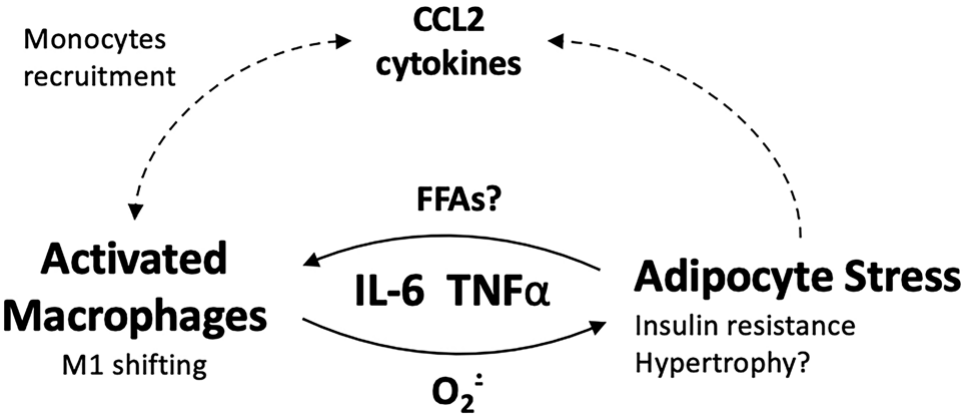
Adipocyte-macrophage interplay in adipose tissue under PM_2.5_ exposure. A tight adipocyte-macrophage interplay is instrumental for the development of WAT and BAT metabolic dysfunction. Macrophage- and adipocyte-released pro-inflammatory cytokines stimulate the recruitment of circulating monocytes. Activated macrophages enhance ROS production and inflammatory response in WAT, increasing adipocyte stress. Stressed/apoptotic adipocytes release pro-inflammatory mediators, sparking a vicious cycle resulting in the development of adipose tissue dysfunction. CCL2, C–C motif chemokine ligand 2; FFAs, fatty free acids; IL-6, interleukin 6; O_2_^−^, superoxide anion; TNFα, tumoral necrosis factor

The protein kinase B (AKT) mediates the transduction of insulin signal [85]. Reduced phosphorylation of AKT^473^ (pAKT) is associated with inflammation and insulin resistance in WAT [86]. In response to PM_2.5_, the expression of pAKT and GLUT-4 significantly decreased in vWAT and BAT of lean [23, 28, 87] and diabetes-susceptible mice (KKay) [26]. In contrast, the administration of antioxidants or the conditional ablation of CCR2 was sufficient to revert these metabolic changes [18, 28]. This and similar evidence suggests that the macrophage-dependant inflammation/ROS production is implicated in the development of insulin resistance in adipose tissue of PM_2.5_-exposed animals [23, 88] (Fig. 2).

The accumulation of macrophages is a recognized mark of adipose tissue inflammation and metabolic dysfunction [30]. In animal models, PM_2.5_ exposure was shown to upregulate vascular adhesion molecules and augmented monocytes in mesenteric blood vessels of both HFD-fed and lean mice [12, 18, 19]. Also, an increased number of activated macrophages in vWAT and BAT is reported by numerous investigations [12, 18, 21, 25–27, 71, 88].

The M1-polarization of macrophages is another feature indicating the inflammatory switch of WAT in PM_2.5_-exposed animals [12, 19]. M1 macrophages accumulate in vWAT of obese animals and generate pro-inflammatory/pro-fibrotic signals [89]. On the contrary, M2 macrophages buffer fluctuations of energy substrates and improve insulin sensitivity [30, 89]. Early findings suggest the M1-shift under PM_2.5_ exposure [12, 19, 90]. For example, in vitro studies showed that high concentrations of PM_2.5_, through ROS-dependent mechanisms, drive the M1-polarization of macrophages in WAT [90]; in addition, PM_2.5_ exposure was shown to suppress the anti-inflammatory IL-10 and the M2-specific markers–macrophage-galactose-type lectin and galactose-N-acetyl-galactosamine–specific lectin–in lean [19] and obese animals [12].

## PM_2.5_ stimulates adipocyte hypertrophy and WAT mass expansion

Adipocyte hypertrophy and WAT mass expansion have been observed in both lean [19, 25, 28, 71, 91] and obese rodents [12, 25] undergoing chronic PM_2.5_ exposure. Observational data in humans substantiate this finding by showing that chronic PM exposure is associated with higher abdominal adiposity [13, 92]. Interestingly, in rodent studies, PM_2.5_-induced WAT mass expansion occurs independently of changes in food intake [25, 28].

**Fig. 3:**
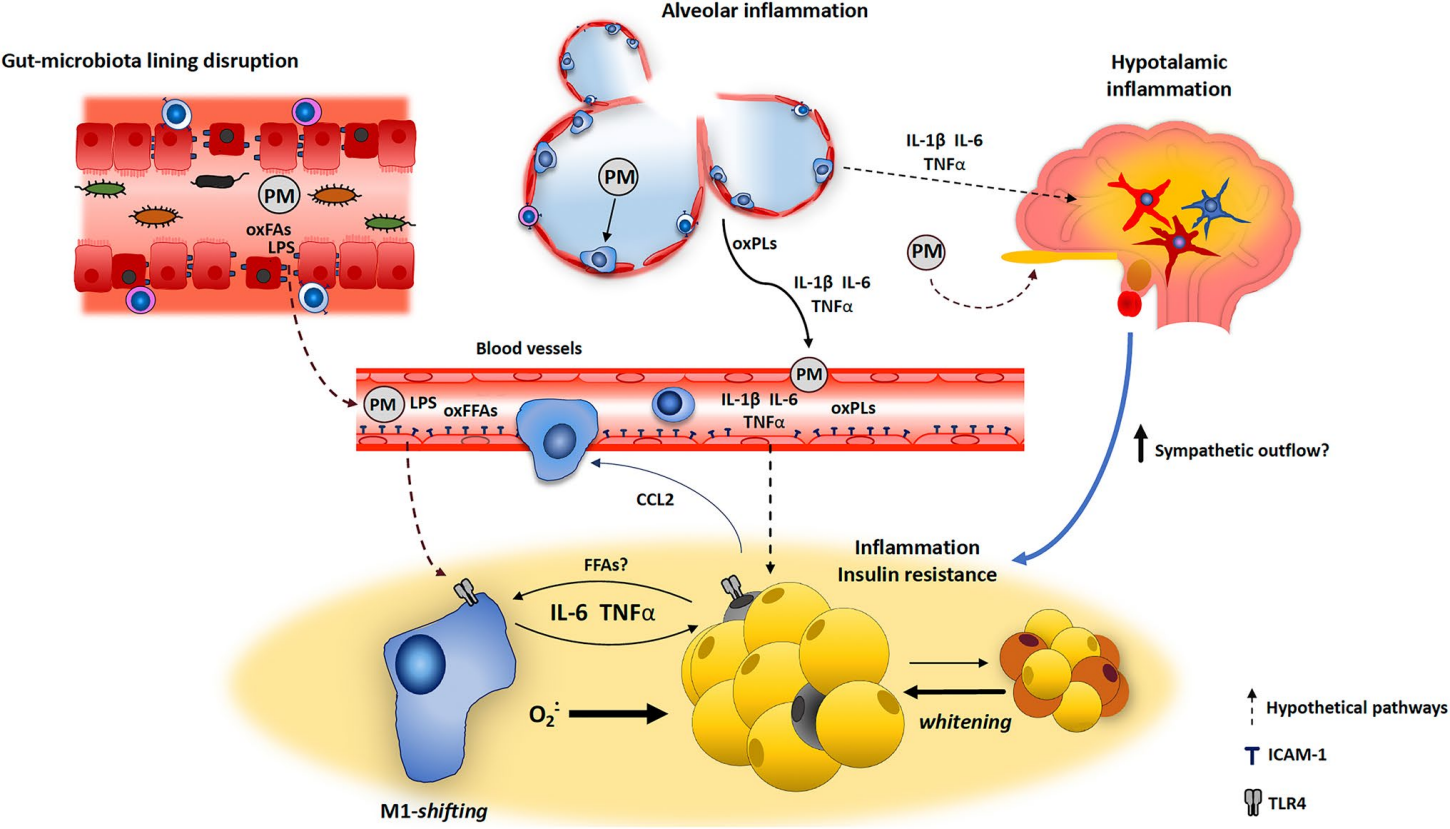
Model showing the potential mechanisms responsible for adipose tissue dysfunction under PM_2.5_ exposure. PM_2.5_-induced alveolar inflammation is responsible for the release of pro-inflammatory mediators into circulation. PM_2.5_ also alters microbiota composition and compromises epithelial integrity, allowing the leakage of pro-inflammatory molecules. Circulating cytokines, LPS, and other byproducts fuel inflammation and insulin resistance in WAT and stimulate the *whitening* of brown adipocytes. PM_2.5_-induced hypothalamic inflammation contributes to increase inflammation and insulin resistance in WAT. CCL2, C–C motif chemokine ligand 2; ICAM, intercellular adhesion molecule 1; LPS, lipopolysaccharide FFAs, free fatty acids. IL-6, interleukin 6; IL-1β, interleukin 1β; O_2_^−^, superoxide anion; OxFFAs, oxidized free fatty acids; OxPLs, oxidized phospholipids; PM, particulate matter; TLR, Toll-like receptor. TNFα, tumoral necrosis factor

Hypertrophy can cause white adipocyte stress and dysfunction [30, 34]. The excessive adipocyte enlargement induces the compression of blood vessels, causing the development of ischemic areas in the context of WAT [34, 93]; chronic hypoxia stimulates the activation of apoptosis and stress/inflammatory pathways, promoting the shifting of WAT toward a dysfunctional phenotype [30, 34, 94] (Figs. 1, 2, and 3).

Although the underlying mechanisms are unclear, early studies suggest that chronic exposure to PM_2.5_ enhances adipo- and lipogenesis in WAT [28, 71]. In PM_2.5_- instilled mice, adiposyte hypertropy and WAT expansion were associated with the upregulation of the pro-adipogenic factors – peroxisome proliferator-activated receptor (PPAR)γ – and – cAMP response element-binding protein (CREB/P)α – [28]. In addition, a significant upregulation of key-enzymes in the synthesis of fatty acid (acetyl-CoA carboxylase, ACC) and TGs synthesis (diglyceride acyltransferase-2, DGAT2) was reported in WAT of PM_2.5_-exposed mice [71]. A decrease in energy expenditure, resulting from hypothalamic dysfunction and BAT *whitening*, can also account for the augmented TGs storage in white adipocytes [20, 21, 25, 26, 71, 95].

On the other hand, adipocyte hypertrophy, in the absence of an energy surplus, suggests the existence of maladaptive mechanisms enhancing TGs accumulation [34]. ER and mitochondrial dysfunction can impair glucose/lipid oxidation, stimulating TGs storage and adipocyte hypertrophy independently from changes in energy intake/expenditure [34, 42, 62, 67]. In PM_2.5_ exposed rodents, for instance, the impairment in mitochondrial biogenesis was associated with WAT mass expansion and adipocyte hypertrophy [25, 28]. Likewise, ER stress was associated with the upregulation of lipogenic markers in WAT in exposed models [71].

One final aspect is that oxidative stress/inflammation can directly inhibit the oxidation of substrates in adipocytes, stimulating TGs storage and hypertrophy [96] (Fig. 2). Supporting this insight, the deletion of the antioxidant transcription factor Nrf-2 was found to exacerbate adipocyte hypertrophy upon PM_2.5_ intratracheal instillation [91]; conversely, the administration of a concentrated antioxidant (e.g., hydroxytyrosol) efficiently reverted adipocytes hypertrophy and vWAT mass expansion [28].

## PM_2.5_ exposure induces the *whitening* of brown adipocytes

Growing evidence reveals that BAT loss and the *whitening* of brown adipocytes occur in animals chronically exposed to PM_2.5_ [23]. Brown adipocytes of PM_2.5_-exposed rodents display a higher number of lipid droplets than filtered-air-exposed controls [17], which indicates an initial phase of *whitening*. PM_2.5_ exposure reduced mitochondrial size and number in BAT of PM_2.5_-exposed [23, 65, 66]. The UCP-1 expression was found to decrease in BAT of PM_2.5_-exposed KKay [26], ApoE-/- [65] and lean mice [17, 23, 28]. Also, chronic exposure to PM_2.5_ suppressed PGC-1α and glucose uptake in BAT [20, 21, 23], while stimulating the expression of markers of *whitening* (e.g., homeobox-C9 and insulin-like growth factor-binding protein-3) [23, 65].

Persistent PM_2.5_ inhalation induces the upregulation of p38, TNFα, and IL-6 [26] and the accumulation of macrophages in BAT [21]. Interestingly, macrophage accumulation was associated with the impairment of glucose uptake, indicating that BAT metabolic dysfunction might be dependent upon inflammation. This occurrence is also suggested by evidence in non-exposed models; macrophages infiltration in WAT is demonstrated to prevent the *browning* of adipocyte precursors [97], and the overexpression of TNFα and CCL2 in BAT is associated with the downregulation of UCP-1 and PGC-1α [98]. In contrast, the inhibition of p38 and JNK promotes the *browning* of white adipocytes [79, 80]. In addition, the suppression of WAT *browning* is probably mediated by the activation of the protein apoptosis signal-regulating kinase 1 (ASK1), a JNK inducer (Fig. 1) [99]. Notably, ASK1 represents a key player in the induction of inflammatory response in alveolar cells upon PM_2.5_ exposure [54].

## Role of other tissues in WAT and BAT dysfunction under PM_2.5_ exposure

In humans and animals, PM_2.5_ exerts its negative effects by perturbing cell and tissue homeostasis. In animal models, 2-μm particles have been shown to reach parenchymatous organs such as the kidney, liver, and spleen, likely transported by alveolar macrophages [53]. Therefore, PM_2.5_ may reach the adipose tissue milieu and exert an in loco toxic effect.

**Fig. 4:**
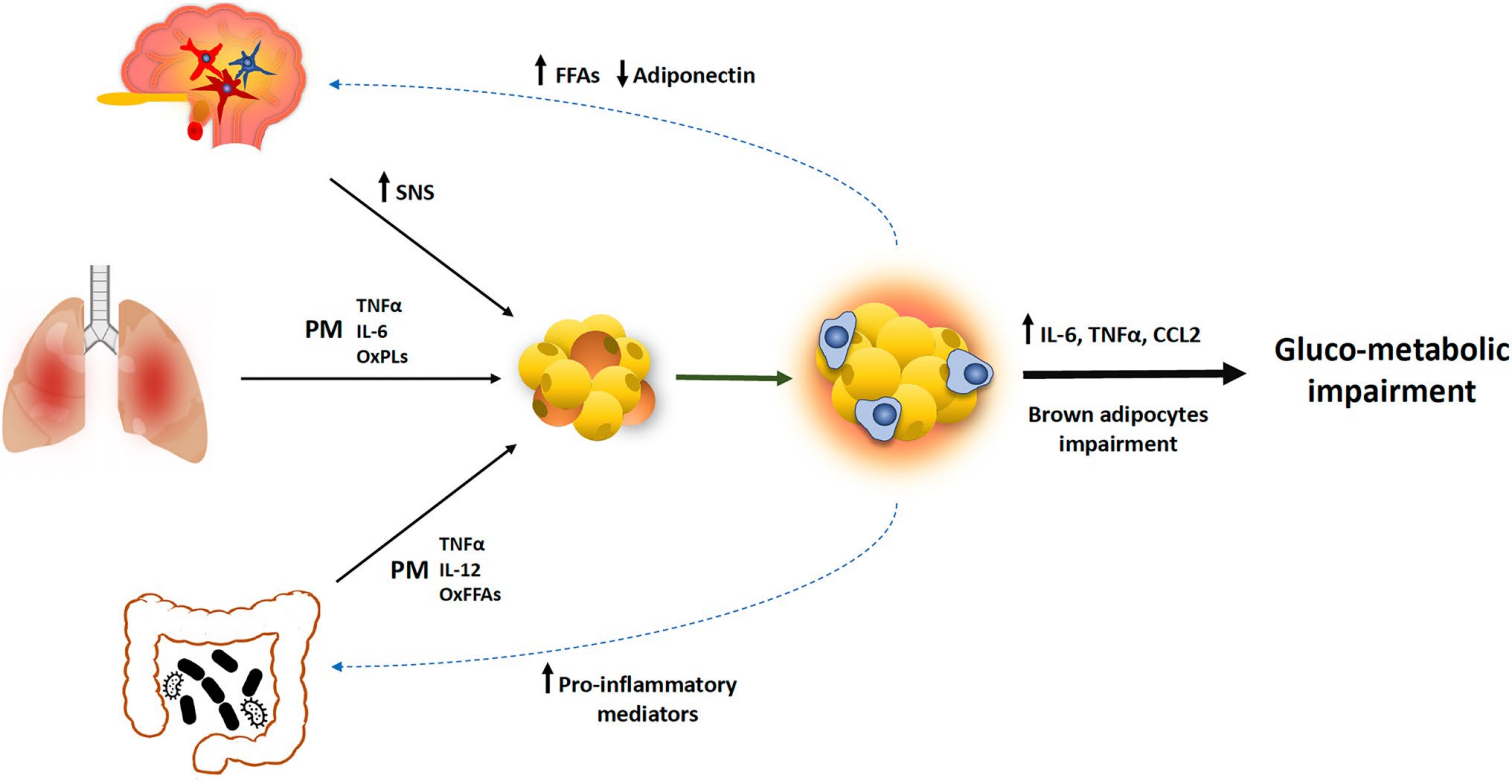
Synoptic scheme showing the potential influence of adipose tissue dysfunction on systemic health under PM_2.5_. Lung- and gut-derived pro-inflammatory mediators and other biomolecules trigger WAT inflammation and metabolic dysfunction. The development of hypothalamic inflammation increases WAT inflammatory response and insulin resistance. Soluble mediators secreted by dysfunctional WAT worsen whole-body metabolic homeostasis and affect the functionality of central/peripheral tissues. BAT, brown adipose tissue; CCL2, C–C motif chemokine ligand 2; IL-6, interleukin 6; IL-12, interleukin 12; OxFFAs, oxidized free fatty acids; OxPLs, oxidized phospholipids; PM, particulate matter; SNS, sympathetic nervous system; TNFα, tumoral necrosis factor

In addition, existing evidence suggests that pro-inflammatory mediators and other biomolecules released by inflamed tissues including the lungs, the GI tract, and the hypothalamus may be responsible for WAT and BAT dysfunction, in PM_2.5_-exposed models (Figs. 3 and 4).

### Alveolar-derived pro-inflammatory mediators may affect BAT and WAT

The activation of alveolar macrophages, following PM_2.5_ inhalation, is responsible for the systemic increase of IL-1β, IL-6, TNFα, and interferon-γ [9, 10, 12, 100]. In experimental models, both the treatment with antioxidants and the induction of SOD expression in the lungs efficaciously inhibited inflammation in periaortic adipose tissue and mitigated insulin resistance in endothelial cells [101, 102], suggesting that lungs inflammation can impair the functionality of distant tissues.

Under chronic PM_2.5_ exposure, lung-released inflammatory mediators can affect adipose tissue metabolic and inflammatory homeostasis. IL-6 was demonstrated to impair insulin signaling and GLUT4 in pre-adipocytes [103]. In mature adipocytes, TNFα inhibited IRS-1 and stimulated IL-6 expression [104, 105]. TNFα also enhances ROS production [96, 106] and suppresses mitochondrial biogenesis and brown adipocytes growth [107, 108]. IL-1β activates NF-κB and inhibits IRS-1 and pAKT, in WAT [109, 110]. Finally, IL-6 and IL-1β can suppress BAT thermogenetic activity and the *browning* of white adipocytes [111–113].

Other organic byproducts derived from the interaction airways-PM_2.5_ may enhance inflammation in adipose tissue. For example, PM-carried endotoxins as well as ROS and organic byproducts released into circulation trigger WAT inflammation by activating the toll-like receptor (TLR) downstream cascade [72, 88, 114] (Figs. 1 and 2).

### Alteration of the gut-microbiota lining can induce WAT and BAT dysfunction

The lower GI tract can be directly exposed to PM, following the consumption of foods carrying high levels of particles [51]. Evidence suggests that up to 10^14^ particles per day can be ingested by consuming a typical western diet, with an overall GI absorption of about 1% [51, 115]. Besides, the muco-ciliary clearance in the upper airways redirects inhaled PM_2.5_ toward the GI tract [116]. Since PM_2.5_ is less readily absorbed [51], it migrates as far as the lower gut [117], interfering with gut-microbiota homoeostasis.

The loss of integrity of colonocytes-microbiota lining represents a potential trigger for WAT dysfunction [118, 119]. Indeed, the leakage of intraluminal LPS, oxidized FFAs, and other pro-inflammatory mediators stimulates adipogenesis and WAT inflammation [96, 106, 118, 120–124]. In addition, gut-derived LPS impairs BAT thermogenic activity by downregulating UCP-1 and the β3-adrenergic signaling [125–127]. On the contrary, the inhibition of mucosal inflammation in the gut is followed by decreased inflammation and insulin resistance in WAT [128].

In experimental models, PM_2.5_ exposure was shown to negatively influence gut-microbiota homeostasis [116, 129]. PM_2.5_ induced a switch in the gut microbiota toward a pro-inflammatory phenotype and augmented the proportion of oxidized FFAs within the gut lumen [130–132]. In mice treated with oral gavage of a PM_2.5_ solution, gut epithelial cells exhibited molecular and morphologic features of dysfunction and death [117]. Also, PM_2.5_ exposure worsened gut permeability by altering the expression of thigh-junctions and by increasing inflammation of the mucosal lining [117, 132–134] (Figs. 2 and 3). Noteworthy, in PM_2.5_ intratracheal-instilled mice, the decrease in gut-microbiota diversity was correlated with white adipocytes hypertrophy and the suppression of UCP-1 in BAT [28].

### Hypothalamic inflammation is associated with insulin resistance and inflammation in WAT

The hypothalamus is a brain structure critical for the integration of multiple peripheral signals to ensure metabolic flexibility and systemic homeostasis. In particular, the mediobasal hypothalamus directly modulates lipogenesis, substrate oxidation and insulin sensitivity of WAT [135, 136].

PM_2.5_ could affect brain functionality by (i) upregulating circulating pro-inflammatory mediators [137]; (ii) inducing inflammation of the olfactory bulb [137]; and (iii) altering gut microbiota ecosystem and the *gut-brain* crosstalk [137, 138].

PM_2.5_ exposure is followed by an intense microglia activation and increased expression of the inhibitor of nuclear factor kappa-B kinase subunit-β (IKKβ), TNFα, and IL-6 in the hypothalamus [139–141]. PM_2.5_ was also shown to upregulate NF-κB and the expression of pro-inflammatory genes in the paraventricular nucleus [141], a region involved in the control of energy balance and sympathetic activation. Interestingly, this neuro-inflammatory setting was accompanied by the accumulation of activated macrophages and the suppression of insulin signaling in vWAT [139]. Of note, either the blockage [139] or the ablation [27] of IKKβ in the hypothalamus effectively prevented macrophages accumulation, the expression of pro-inflammatory mediators, and insulin resistance in vWAT. Altogether, these findings strongly suggest that WAT dysfunction, under PM_2.5_, could be driven by hypothalamic inflammation (Fig. 3) [139–145].

## Conclusion and future directions

Current animal studies demonstrate that PM_2.5_ exposure significantly increases inflammation in both WAT and BAT. Mitochondrial impairment, insulin resistance, and the *whitening* of brown adipocytes indicate the development of metabolic dysfunction.

While the underlying mechanisms are not fully elucidated, PM_2.5_-driven alterations in the lungs, the gut, and the hypothalamus appear to play an important role in driving inflammation and dysmetabolism in both WAT and BAT.

Noteworthy, the perturbation of adipose tissue homeostasis, under PM_2.5_ exposure, may worsen systemic cardio-metabolic health and the functionality of central/peripheral organs (Fig. 4).

Further investigations should be aimed to (i) investigate the accumulation of PM_2.5_ in adipose tissue and mechanisms of damage in adipocytes; (ii) characterize the activation of lipolysis/lipogenesis as well as the release of adipose tissue-specific mediators (adiponectin, FFAs, diacylglycerols, ceramides); and (iii) estimate the effect of adipose tissue dysfunction on gluco-metabolic balance in exposed models.

## Literature search methods

The research has been carried out on Medline, Scopus and Embase by restricting the language to English. The search strategy was assessed by alternatively combining the keywords “adipose tissue,” “adipocyte,” “metabolism,” “obesity” with “PM,” “fine particulate matter,” and “air pollution.” Data strictly pertaining to experimental evidence investigating the effect of fine particulate matter (PM; 2.5 μm, in diameter) on WAT and BAT have been retrieved. In order to describe the pathophysiological mechanisms underlying BAT and WAT dysfunction, we have restricted our research to controlled studies on animals undergoing direct exposure in a confined chamber or intratracheal instillation (a validated method to replicate airborne PM exposure in animal models) [146] of PM_2.5_.

## Data Availability

Not applicable.

## Abbreviations

ACC: Acetyl-CoA Carboxylase
AKT: Protein kinase B
AMPK: AMP-activated protein kinase
ASK1: Apoptosis signal-regulating kinase 1
BAT: Brown adipose tissue
CCL2: C-C motif chemokine ligand 2
CCR-2: C-C chemokine receptor type 2
CREB/Pα: CAMP response element-binding protein
DGAT2: Diglyceride acyltransferase-2
ER: Endoplasmic reticulum
FFAs: Fatty free acids
GI: Gastrointestinal
GLUT4: Glucose transporter 4
BiP: Binding immunoglobulin protein
HFD: High fat diet
HSP72: Heat shock protein 70 kilodaltons
IKK: Inhibitor of nuclear factor kappa-B kinase subunit-β
IL-1β: Interleukin 1 beta
IL-10: Interleukin 10
IL-6: Interleukin 6
JNK: C-Jun N-terminal kinase
LPS: Lipopolysaccharide
NF-κB: Nuclear factor kappa-light-chain-enhancer of activated B cells
Nrf-2: Nuclear factor erythroid 2-related factor 2
OxPLs: Oxidized phospholipids
p38MAPK: P38 Mitogen-activated protein kinases
PAHs: Polycyclic aromatic hydrocarbons
PGC-1α: Peroxisome proliferator-activated receptor gamma coactivator 1
PM: Particulate matter
PPARγ: Peroxisome proliferator-activated receptor
ROS: Reactive oxygen species
SOD: Superoxide dismutase
TGs: Triglycerides
TLR: Toll-like receptor
TNF: Tumor necrosis factor
UCP: Uncoupling protein
UPR: Unfolded protein response
WAT: White adipose tissue
