# Correction to: White and brown adipose tissue functionality is impaired by fine particulate matter (PM_2.5_) exposure

**DOI:** 10.1007/s00109-022-02200-8

**Published:** 2022-04-27

**Authors:** Lucio Della Guardia, Andrew C. Shin

**Affiliations:** 1 Department of Biomedical Sciences for Health, Università Degli Studi Di Milano, via Fratelli Cervi 93, 20090 Segrate, Milano, Italy; 2 Department of Nutritional Sciences, College of Human Sciences, Texas Tech University, Lubbock, TX USA

## Correction to: Journal of Molecular Medicine 10.1007/s00109-022-02183-6

The published article should be Open Access.

The Original article has been corrected.

